# Surgical insights into meningiomas en plaque: A rare case of frontal tumor resection and its clinical implications

**DOI:** 10.1016/j.ijscr.2024.110796

**Published:** 2024-12-28

**Authors:** Saja Karaja, Ayham Qatza, William Borghol, Mulham Alkassem, Manar Assaf, Habib Jarbouh

**Affiliations:** a Faculty of Medicine, Hama University, Hama, Syria; b Department of Neurosurgery, Hama National Hospital, Hama, Syria; c Department of Pathology, Damascus University, Damascus, Syria

**Keywords:** Meningiomas, Meningiomas en plaque, Hemiplegia, Proptosis

## Abstract

**Introduction:**

Meningiomas en Plaque (MEP) represents a rare subtype, typically affecting females and seen in the fifth decade of life, with proptosis as a common presenting symptom, posing both diagnostic and surgical challenges.

**Case presentation:**

A 55-year-old right-handed male presented with right hemiplegia, headache, vomiting, and frequent seizures. Neurological examination showed reduced visual acuity and right-sided exophthalmos. MRI revealed a large, enhancing extra-axial dural-based lesion in the left frontal hemisphere, prompting surgical intervention. A bicoronal incision allowed for a left frontal craniotomy and tumor resection, including ligation of the superior sagittal sinus. Microscopic analysis showed spindle cell proliferation with whorled patterns. Postoperatively, the patient had an uneventful recovery and was discharged after three days, reporting resolution of symptoms during follow-up.

**Clinical discussion:**

The unique combination of the tumor's location, clinical manifestations, the patient's gender and age, along with the overall rarity of MEP, underscores the exceptional nature and significance of this case report.

**Conclusion:**

This report emphasizes the need for a comprehensive understanding of the diverse manifestations of this rare subtype, underscoring the challenges associated with its diagnosis and treatment, and contributing valuable insights to the existing knowledge base regarding this unique pathology.

## Highlights

• Meningiomas en Plaque (MEP) account for 2% to 9% of .meningiomas, highlighting their rarity in clinical practice
• Predominantly affects females in their fifth decade, although .cases in males exist, as shown in this report
• MEP typically arises in the sphenoorbital region but can also .affect temporal and cerebral convex areas
• Effective treatment requires collaboration among neurosurgeons, radiologists, and oncologists for optimal .patient care
• Increased knowledge of MEP's diverse manifestations is crucial for timely diagnosis and intervention in clinical .settings

## 1 Introduction

Meningiomas are the most prevalent primary brain tumors, with an age-adjusted incidence of 7.8 per 100,000 individuals [1]. Meningiomas en Plaque (MEP) is a rare subtype, accounting for only 2 % to 9 % of all meningiomas [2]. MEP's defining characteristic is its unique “carpet-like” invasion of adjacent bone [1]. Commonly observed in the sphenoorbital region, MEP can also invade the temporal region, cerebral convex area, and foramen magnum [3]. Proptosis is usually the most common presenting symptom of MEP [4]. Radiological evaluation utilizing computed tomography (CT) scans and magnetic resonance imaging (MRI) is crucial in establishing the possibility of MEP [3]. The unusual radiological characteristics of MEP pose a significant diagnostic challenge. Furthermore, the propensity of MEP to invade bony structures and intricately interweave within nearby fissures and foramina presents significant surgical difficulty [1]. This paper describes a rare case of MEP, which appears to be unique in terms of the location of the tumor and clinical manifestations, including right hemiplegia and recurrent seizures, and provides a thorough overview of the patient's condition, diagnostic approach used, surgical strategies, and outcomes, hoping to raise awareness of this rare entity.

This case report is aligned with SCARE criteria, which contributes to its transparency and overall quality [5].

## 2 Case presentation

**Fig. 1:**
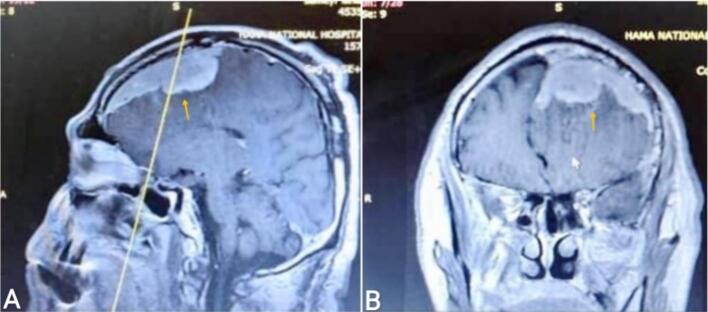
Brain magnetic resonance imaging (MRI) with contrast. The sagittal section of T1 sequence (image A) and coronal section of T1 sequence (image B) both demonstrate intensely enhancing extra-axial dural-based lesion along the frontal left hemisphere (yellow arrow).

**Fig. 2:**
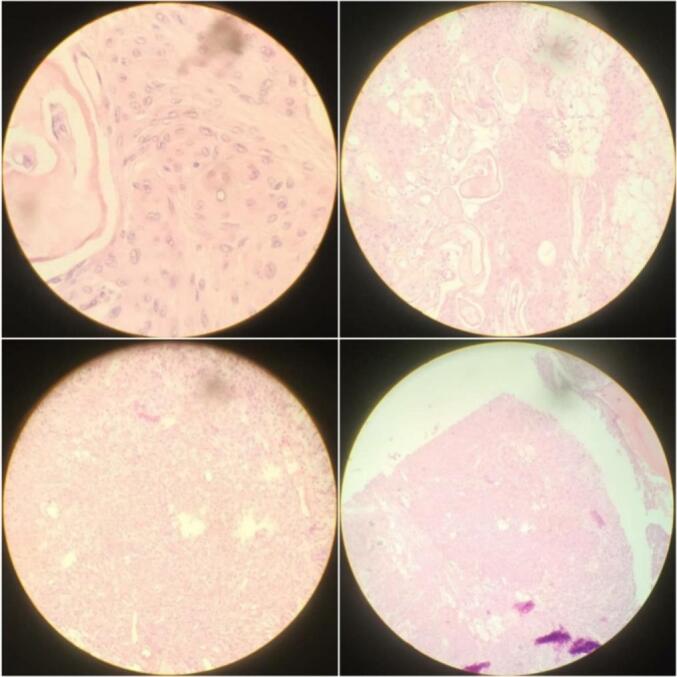
Microscopic examination of serial sections reveals diffuse proliferation of spindle cells with oval nuclei and pale cytoplasm; these cells form whorls with calcified stroma.

**Fig. 3:**
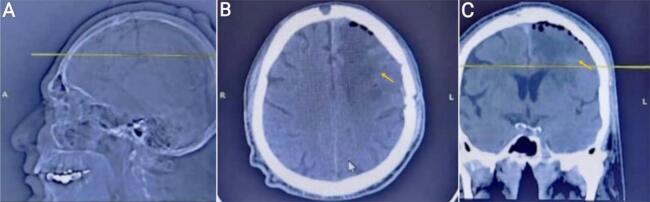
Postoperative computed tomography (CT) tissue window showed complete resolution of the space-occupying lesion with mild cerebral edema at the surgical site (yellow arrow). (A: Sagittal sequence, B: Axial sequence, C: Coronal sequence).

A 55-year-old right-handed male patient presented to the emergency department with a one-month history of right hemiplegia, headache, vomiting, and more than three seizures daily. The patient's medical history involves no previously diagnosed disorders or prescribed medications. Comprehensive physical and ophthalmological examination indicated stable hemodynamic and neurological conditions, with significant visual acuity loss and pronounced right-sided exophthalmos. The physical examination of other systems and the laboratory investigations were normal. Initial brain MRI with contrast revealed an intensely enhancing extra-axial dural-based lesion along the left frontal hemisphere, causing mass effect, resultant vasogenic edema, and midline shift to the right [Fig. 1]. Providing the neurologic symptomatology and the radiologic location of the mass, surgical removal was recommended. The patient's written consent was obtained, and the medical consultations prior to the procedure indicated no objections to the surgery. The surgery was done by a specialist in neurosurgery, which involved a standard bicoronal incision, sparing the pericranium, and elevating the skin flap while preserving the superficial temporal arteries. A left frontal craniotomy was performed, including ligation and excision of the anterior third of the superior sagittal sinus with the dura mater. The tumor was meticulously removed using a surgical microscope, with coagulation of feeding vessels. Additionally, a dura graft was conducted using the patient's own scalp peritoneum. Microscopic examination of serial sections revealed diffuse proliferation of spindle cells with oval nuclei and pale cytoplasm; these cells form whorls with calcified stroma [Fig. 2]. Based on the aforementioned histological findings, the diagnosis of a Grade I MEP was made. After surgery, CT scans showed complete resolution of the space-occupying lesion with mild cerebral edema at the surgical site [Fig. 3]. The patient's recovery after the surgery was uneventful, and he was discharged from the hospital after three days. The patient has been on a regular follow-up. At the last review, he was asymptomatic, and his physical examination revealed disappearance of headache, right hemiplegia, and seizures.

## 3 Discussion

Meningiomas are classified as the most common primary neoplasm of the central nervous system, accounting for about 37.6 % of them and about half of all benign brain tumors [1,6]. MEP is a rare type of meningioma characterized by carpet-like lesions in the dura mater that lead to hyperostosis and invasion of the adjacent bone with extracranial extension into the orbit and soft tissues, unlike typical meningiomas [1,7]. This is where the uniqueness of this case begins, because our patient was a male, 55 years old, which contradicts the traditional epidemiology of MEP, which shows that females are 3 to 6 times more affected than males and that the most common age of occurrence is between 40 and 50 years [4,7]. MEP commonly occurs at the cranial base and is distinguished more by its clinical and biological behavior than by histological appearance. These tumors often cause hyperostosis in the skull at the tumor site, mainly within the middle cranial fossa, especially affecting the sphenoid wing, with rare occurrences in other regions such as the cranial vault's membranous bone [8]. Contrary to what is mentioned in the medical literature, MEP in this case is located in the anterior cranial fossa on the left frontal side without the appearance of hyperostosis in the skull at the tumor site. The clinical manifestations of this tumor depend mainly on its location, determining which of the neural structures are affected and the extent of tumor spread [1]. The spheno-orbital region is the most common location for MEP, which can manifest clinically through symptoms including proptosis, diplopia, headache, eyelid ptosis, and visual field damage associated with optic canal stenosis [1,4]. What makes the case even more unique is that our patient did not suffer from symptoms similar to the traditional symptoms described, as the main complaint was right hemiplegia with recurrent seizures without a history of epilepsy in the patient, and these are different signs that do not refer to the traditional location of MEP. Both MRI and CT are the initial diagnostic imaging. MRI enables the study of dura involvement in the tumor, which usually shows a clear contrast enhancement after gadolinium injection at T1, while CT plays a prominent role in studying the bone and hyperostosis adjacent to the tumor using the bone window in CT [2]. The biological behavior and growth pattern of MEP exhibit considerable variability, ranging from mild and slowly progressive to severe and rapidly expanding [4]. In addition, meningiomas are categorized into three grades (1–3) based on histological and molecular characteristics. Grade 1 tumors are less aggressive, while grades 2 and 3 exhibit increased concern. Grade 2 shows more mitotic figures, brain tissue invasion, and specific histological subtypes (choroid or clear cell kinds). Grade 3 is marked by sarcoma, carcinoma, or melanoma-like appearances and telomerase reverse transcriptase (TERT) promoter mutations [9]. According to the World Health Organization (WHO), MEP usually follows the classification grade 1, meaning that they are benign tumors despite local invasion [7]. Histological examination of MEP shows that meningothelial cells invade the skull bones in addition to the expansion of the Haversian canals of the bone, which increases the thickness of the bone [1,7]. Secondary hyperostosis is also observed, with rare instances of vascular and muscular invasion reported [1]. The patient in the present case was suspected of having MEP through the use of MRI and CT, which was later confirmed by microscopic examination of the resected specimen. Although complete surgical excision is the standard intervention for MEP, the challenge of extensive local invasion adjacent to important structures may necessitate partial resection, which carries a high risk of recurrence [7]. Furthermore, comprehensive analysis of preoperative imaging is crucial for determining the optimal surgical strategy for these lesions, focusing on achieving wide margins around hyperostotic bone without risking critical neurovascular structures [1]. Therefore, it is imperative to have a clear understanding of these conditions to devise an appropriate surgical approach. Despite exploring multiple approaches such as transzygomatic, transcranial–transmalar, and cranio-orbital methods, the pterional craniotomy is the most commonly employed for sphenoid wing tumors, with orbital and zygomatic modifications applied as necessary [1]. In the present case, an unusual surgical approach was conducted, where the surgical excision was performed using a high-precision surgical microscope with coagulation of the feeding vessels by performing a left frontal craniotomy to accommodate the tumor site, which contributed to the complete removal of the tumor. Advances in microsurgical techniques have improved outcomes, making complete and early surgical excision the primary treatment for MEP, with adjuvant radiotherapy considered for partial resections [1].

## 4 Conclusion

This case of MEP exemplifies the rarity of this subtype, emphasizing the critical importance of broadening clinicians' understanding of its diverse clinical manifestations and its diagnostic and surgical challenges. In addition, it underscores the need for continued research and awareness in the field to effectively address the complexities associated with the diagnosis and treatment of MEP, particularly in atypical presentations. Further studies and reports of similar cases can contribute to an enhanced comprehension of MEP, ultimately improving patient outcomes and advancing medical approaches for this distinctive subtype of meningiomas.

## Abbreviations

MEP: Meningiomas en Plaque
CT: Computed tomography
MRI: Magnetic resonance imaging
TERT: Telomerase reverse transcriptase
WHO: World health organization

## Ethics approval and consent to participate

Ethics clearance was not necessary since the University waives ethics approval for publication of case reports involving no patients' images, and the case report is not containing any personal information. The ethical approval is obligatory for research that involves human or animal experiments.

## Guarantor

Ayham Qatza

## Research registration number

This case report is not a first time of reporting, new device or surgical technique. So I would not need a Research Registry Unique identifying number (UIN).

## Consent for publication

Written informed consent was obtained from the patient for publication of this case report and any accompanying images. A copy of the written consent is available for review by the Editor-in-Chief of this journal on request.

## Declaration of Generative AI and AI-assisted technologies in the writing process

None.

## Funding

The author(s) received no financial support for the research, authorship, and/or publication of this article.

## Author contribution

Saja Karaja: Writing – review & editing, Writing – original draft, Data curation.

Ayham Qatza: Writing – review & editing, Writing – original draft.

William Borghol: Writing – review & editing, Writing – original draft.

Mulham Alkassem: Writing – review & editing, Writing – original draft.

Manar Assaf: Writing – review & editing, Supervision.

Habib Jarbouh: Writing – review & editing, Supervision.

Ayham Qatza: Submitted the final manuscript.

All authors read and approved the final manuscript.

## Conflict of interest statement

The authors declare that they have no known competing financial interests or personal relationships that could have appeared to influence the work reported in this paper.

## Data Availability

Data sharing not applicable to this article as no datasets were generated or analyzed during the current study.
